# Plasma Epstein-Barr Virus DNA load for diagnostic and prognostic assessment in intestinal Epstein-Barr Virus infection

**DOI:** 10.3389/fcimb.2024.1526633

**Published:** 2025-01-07

**Authors:** Chunxiang Ma, Mingshan Jiang, Jiaxin Li, Zhen Zeng, Yushan Wu, Rui Cheng, Hao Lin, Jiangmei Pang, Fang Yin, Yongbin Jia, Lili Li, Hu Zhang

**Affiliations:** ^1^ Department of Gastroenterology, West China Hospital, Sichuan University, Chengdu, China; ^2^ Centre for Inflammatory Bowel Disease, West China Hospital, Sichuan University, Chengdu, China; ^3^ Lab of Inflammatory Bowel Disease, Frontiers Science Center for Disease-Related Molecular Network, West China Hospital, Sichuan University, Chengdu, China

**Keywords:** Epstein-Barr virus DNA load, diagnosis, prognosis, inflammatory bowel diseases, primary intestinal lymphoproliferative diseases, intestinal Epstein-Barr Virus infection

## Abstract

**Background:**

The prospective application of plasma Epstein-Barr virus (EBV) DNA load as a noninvasive measure of intestinal EBV infection remains unexplored. This study aims to identify ideal threshold levels for plasma EBV DNA loads in the diagnosis and outcome prediction of intestinal EBV infection, particularly in cases of primary intestinal lymphoproliferative diseases and inflammatory bowel disease (IBD).

**Methods:**

Receiver operating characteristic (ROC) curves were examined to determine suitable thresholds for plasma EBV DNA load in diagnosing intestinal EBV infection and predicting its prognosis.

**Results:**

108 patients were retrospectively assigned to the test group, while 56 patients were included in the validation group. Plasma EBV DNA loads were significantly higher in the intestinal EBV infection group compared to the non-intestinal EBV infection group (Median: 2.02 × 10^2^ copies/mL, interquartile range [IQR]: 5.49 × 10^1^-6.34×10^3^ copies/mL versus 4.2×10^1^ copies/mL, IQR: 1.07 ×10^1^-6.08×10^1^ copies/mL; P < 0.0001). Plasma EBV DNA levels at 9.21×10^1^ and 6.77×10^1^ copies/mL proved beneficial for the identification and prognostication in intestinal EBV infection, respectively. Values of 0.82 and 0.71 were yielded by the area under the ROC curve (AUC) in the test cohort, corresponding to sensitivities of 84.38% (95% confidence interval [95%CI]: 68.25%-93.14%) and 87.5% (95%CI: 69%-95.66%), specificities of 83.33% (95%CI: 64.15%-93.32%) and 68.09% (95%CI: 53.83%-79.6%), positive predictive values (PPV) of 87.1% (95%CI: 71.15%-94.87%) and 58.33% (95%CI: 42.2%-72.86%), and positive likelihood ratios (LR^+^) of 5.06 and 2.74 in the validation cohort, respectively. Furthermore, a plasma EBV DNA load of 5.4×10^2^ copies/mL helped differentiate IBD with intestinal EBV infection from primary intestinal EBV-positive lymphoproliferative disorders (PIEBV+LPDs), achieving an AUC of 0.85 within the test cohort, as well as 85% sensitivity (95%CI: 63.96%-94.76%), 91.67% specificity (95%CI: 64.61%-99.57%), 94.44% PPV (95%CI: 74.24%-99.72%), and an LR^+^ of 10.2 in the validation cohort.

**Conclusions:**

Plasma EBV DNA load demonstrates notable potential in distinguishing between different patient cohorts with intestinal EBV infection, although its sensitivity requires further optimization for clinical application.

## 1 Introduction

Epstein-Barr virus (EBV), a pervasive herpesvirus, is particularly recognized for its oncogenic potential in various malignancies and its association with autoimmune diseases (Young et al., 2016). The pathogenic role of EBV has been well established in multiple diseases, such as infectious mononucleosis (Lennon et al., 2015), Burkitt lymphoma (BL) (López et al., 2022), and nasopharyngeal cancer (NPC) (Wong et al., 2021). However, research into the impact of EBV on intestinal diseases, particularly inflammatory bowel disease (IBD), together with primary intestinal lymphoproliferative diseases (PILPDs), is just beginning (Rizzo et al., 2017; Dang et al., 2023; Chen L. et al., 2024). IBD is a multifaceted disease characterized by persistent inflammation within the gastrointestinal tract, which is often exacerbated by opportunistic infections (Cao et al., 2022; Zeng et al., 2023; Li et al., 2024; Pu et al., 2024). Evidence highlights that the incidence of positive EBV DNA detection from intestinal resection specimens in patients with IBD is 55%-76%, which is markedly higher than the 19% observed in a non-IBD group (Xu et al., 2020). Furthermore, EBV infection is intricately linked to clinical manifestations (Dimitroulia et al., 2013), therapeutic responses (Pezhouh et al., 2018; Chen Y. et al., 2024), surgical interventions (Hosomi et al., 2018), and lymphoma incidence (Nissen et al., 2015; de Francisco et al., 2018) in IBD patients. Moreover, the diagnosis of intestinal diseases is significantly influenced by EBV infection. PILPDs encompass a spectrum of diseases characterized by abnormal lymphocyte proliferation in the intestine, with manifestations ranging from benign to malignant; notably, aggressive PILPDs are linked to an extremely high risk of mortality (Cohen et al., 2009; Montes-Mojarro et al., 2020). Our previous study revealed that 67% of 12 patients with primary intestinal EBV-positive lymphoproliferative disorders (PIEBV+LPDs) were initially diagnosed with IBD, and half of the 12 patients ultimately succumbed to PIEBV+LPDs (Wang Z. et al., 2018). This underscores the fact that overlooking intestinal EBV infection can lead to fatal outcomes. It is equally important to acknowledge that the gold standard for diagnosing EBV infection is histological analysis using EBV-encoded small RNAs *in situ* hybridization (EBER-ISH) (Weiss and Chen, 2013). However, given the anatomical location of the intestine, the intestinal EBER-ISH test requires invasive procedures, such as endoscopy or surgery. Therefore, the clinical significance of other noninvasive, dependable, and accessible methods for diagnosing intestinal EBV infection needs to be explored.

Measuring peripheral EBV DNA levels shows superiority in simplicity and non-invasiveness for diagnosing and surveilling EBV infection when compared with the histological EBER-ISH test. However, the value of the test is significantly influenced by the choice of peripheral blood components analyzed, such as whole blood, plasma, and peripheral blood mononuclear cells (PBMCs), which may affect sensitivity and specificity. Owing to the latent characteristics of EBV, research proved that measuring EBV DNA levels in plasma has advantages over measuring them in whole blood and PBMCs as indicators of active replication of the virus, thereby improving the diagnostic and prognostic power of diseases associated with EBV (Tsai et al., 2015; Kanakry et al., 2016; Ludvigsen et al., 2023). For instance, the diagnostic sensitivity of a detectable plasma EBV DNA load can reach up to 90% in BL (Hohaus et al., 2011) and as high as 93.2% in NPC (Lou et al., 2023). Furthermore, plasma EBV DNA levels showed considerable predictive power for the outcomes of these diseases (Hui et al., 2020; Qiu et al., 2020). However, in EBV-associated intestinal diseases, although a study reported that IBD patients infected with intestinal EBV exhibited heightened concentrations of EBV DNA within their peripheral whole blood (Xu et al., 2020), no study has employed plasma EBV DNA load, an indicative marker of active infection, to establish definitive thresholds for aiding diagnostic and prognostic assessment of intestinal EBV infection.

Therefore, we conducted a retrospective investigation to define the diagnostic and prognostic cut-off values for intestinal EBV infection based on plasma EBV DNA loads. This approach will facilitate the differential diagnosis of intestinal EBV infections in the clinical setting.

## 2 Materials and methods

### 2.1 Study population and design

This retrospective investigation was performed at the Gastroenterology Department of West China Hospital between January 2013 and January 2024. Initially, patients with positive plasma EBV DNA loads and intestinal diseases were screened through electronic medical record system, followed by verification through the pathology system to confirm whether they had also undergone intestinal EBER-ISH test. Patients with intestinal diseases who tested positive for peripheral blood EBV DNA and completed the intestinal EBER-ISH test were included in the study, while those who did not complete both tests, had incomplete clinical data, or had unclear diagnoses were excluded. Immunomodulator usage was defined as treatment with steroids, immunosuppressants, or biological agents within three months prior to the intestinal EBER-ISH test, and intestinal EBV infection was defined as positive EBER-ISH test in the intestine. The diagnosis of IBD followed recognized criteria, including typical clinical presentations, endoscopic findings, radiological assessments, and histological results (Gomollón et al., 2017; Magro et al., 2017). The diagnostic criteria for PILPDs require the presence of gastrointestinal symptoms and confirmation of abnormal proliferation of lymphocytes within the intestinal tissues through pathology, while excluding primary lymphoproliferative diseases at other sites (Wang Z. et al., 2018). On this basis, a positive result for intestinal EBER-ISH test was defined as PIEBV+LPDs, while a negative result was defined as primary intestinal non-EBV-associated lymphoproliferative diseases (PINEBV+LPDs). Data from the electronic medical record system were also collected to analyze clinical data, such as sex, age, plasma EBV DNA load, and other factors. Furthermore, a follow-up on the prognosis of all patients was conducted, where events such as intestinal resection or death within six months after undergoing intestinal EBER-ISH test were defined as fatal events, while all other outcomes were considered benign events. Ethics approval for the present investigation was obtained from the ethics board of West China Hospital. (Number: 2023-22).

### 2.2 EBV test

As directed by the manufacturer, quantitative measurement of EBV DNA in the plasma was performed using an EBV detection kit with polymerase chain reaction (PCR) fluorescence (Shen Xiang Gene Co.). The EBER gene was amplified using a Bio-Rad CFX96 PCR instrument. A Ct value ≤39 was interpreted as positive, and a standard curve was used to calculate the quantity of EBV DNA copies. EBER-ISH was used to investigate intestinal EBV infection, and the EBV-encoded small RNAs (EBER) peptide nucleic acid probe was sourced from ZSGB-BIO (China). Specifically, tissues from intestinal biopsies of patients were deparaffinized, rehydrated, and permeabilized with proteinase K, followed by overnight hybridization at 37°C with digoxigenin-labeled EBER probes. After incubation with horseradish peroxidase-conjugated anti-digoxigenin antibody, EBER-positive cells were identified by diaminobenzidine staining and exhibited brown-stained nuclei. These cells were quantitatively scored by two experienced pathologists in high-power field (HPF).

### 2.3 Statistical analysis

Statistical evaluation was performed using SPSS and GraphPad Prism (version 26.0 and 9.5, respectively). This investigation of the demographic and basic features of the patients utilized both frequency distributions and descriptive statistical methods. To compare patient characteristics among different cohorts, either the chi-squared hypothesis evaluation or the exact significance test using Fisher’s exact test was employed, contingent on the distributional features inherent in these data. To evaluate the role of plasma EBV DNA load in the diagnostic and prognostic assessment of intestinal EBV infection, a stepwise analysis was conducted using both the train and validation cohorts. In the first stage, receiver operating characteristic (ROC) curves were utilized to analyze plasma EBV DNA concentrations for diagnosing intestinal EBV infection and predicting its prognosis. These optimal cutoff values were determined based on the maximum Youden’s index, which maximizes the sum of sensitivity and specificity (Youden’s index = sensitivity + specificity-1). This approach ensures the identification of the threshold that achieves the best trade-off between true-positive and true-negative rates for each cohort. In the next stage, the performance of these cutoff values was assessed in the validation cohort by calculating sensitivity, specificity, positive predictive value (PPV), negative predictive value (NPV), positive likelihood ratio (LR^+^), and negative likelihood ratio (LR^-^). These metrics were used to confirm the robustness of the cutoff values derived from the train cohort. Statistical significance was represented by a p-value of < 0.05.

## 3 Results

### 3.1 Clinical characteristics among train cohort

Our investigation began with the identification of 213 patients diagnosed with intestinal diseases and tested positive for plasma EBV DNA. After excluding patients with unclear diagnoses and incomplete clinical data, the remaining patients were randomly assigned in a 2:1 ratio, resulting in a cohort of 108 patients in the train group and 56 patients in the validation group (**Supplementary Figure 1**; **Supplementary Table 1**). The train cohort consisted of 60 patients (55.56%) who tested negative for intestinal EBER-ISH and 48 patients (44.44%) who tested positive for intestinal EBER-ISH. The median age of the EBER-negative group was 42 years, which was comparable to that of the EBER-positive group (median age, 44 years). Moreover, neither sex distribution nor disease course demonstrated notable differences between the two groups. Additionally, the EBER-positive group did not exhibit a significantly higher frequency of immunomodulator use within three months preceding the intestinal EBER-ISH test than the EBER-negative group. However, a disparity in disease type was observed between the two groups. The EBER-positive group predominantly consisted of patients with PILPDs (50%), followed by IBD (47.9%) and other diseases (2.1%), whereas IBD was the most prevalent disease in the EBER-negative group (76.7%). The symptoms in both groups also differed significantly in clinical terms, with the EBER-positive group showing a higher incidence of fever and hematochezia than the EBER-negative group (**Table 1**).

**Table 1:** Clinical data of patients in the training and validation cohort.

Characteristics	Test cohort			Validation cohort		
	Intestinal EBER-negative group (N=60)	Intestinal EBER-positive group (N=48)	*P* value	Intestinal EBER-negative group (N=24)	Intestinal EBER-positive group (N=32)	*P* value
Age (year), median ± SD	42 ± 17	44 ± 15	0.431	47 ± 16	41 ± 16	0.815
Male, n (%)	40 (66.7%)	28 (58.3%)	0.373	14 (58.3%)	22 (68.8%)	0.421
Disease duration (months), median ± SD	40 ± 48	44 ± 86	0.7696	32 ± 43	33 ± 60	0.938
IMM use within three months prior to intestinal EBER-ISH test, n (%)	14 (23.3%)	14 (29.2%)	0.492	5 (20.8%)	8 (25%)	0.715
IBD	46 (76.7%)	23 (47.9%)	0.079	18 (75%)	12 (37.5%)	0.125
PILPDs	7 (11.7%)	24 (50%)		3 (12.5%)	20 (62.5%)	
Other diseases	7 (11.7%)	1 (2.1%)		3 (12.5%)	0 (0%)	
Fever	10 (16.7%)	26 (54.2%)	<0.0001	7 (29.2%)	20 (62.5%)	0.013
Abdominal pain	41 (68.3%)	38 (79.2%)	0.207	17 (70.8%)	21 (65.6%)	0.68
Diarrhea	32 (53.3%)	31 (64.6%)	0.239	13 (54.2%)	20 (62.5%)	0.53
Haematochezia	31 (51.7%)	35 (72.9%)	0.024	15 (62.5%)	24 (75%)	0.314
Weight loss	34 (56.7%)	32 (66.7%)	0.289	11 (45.8%)	19 (59.4%)	0.315

SD, standard deviation; IMM, immunomodulators.

### 3.2 Distribution of plasma EBV DNA loads in intestinal EBV infection

**Figure 1:**
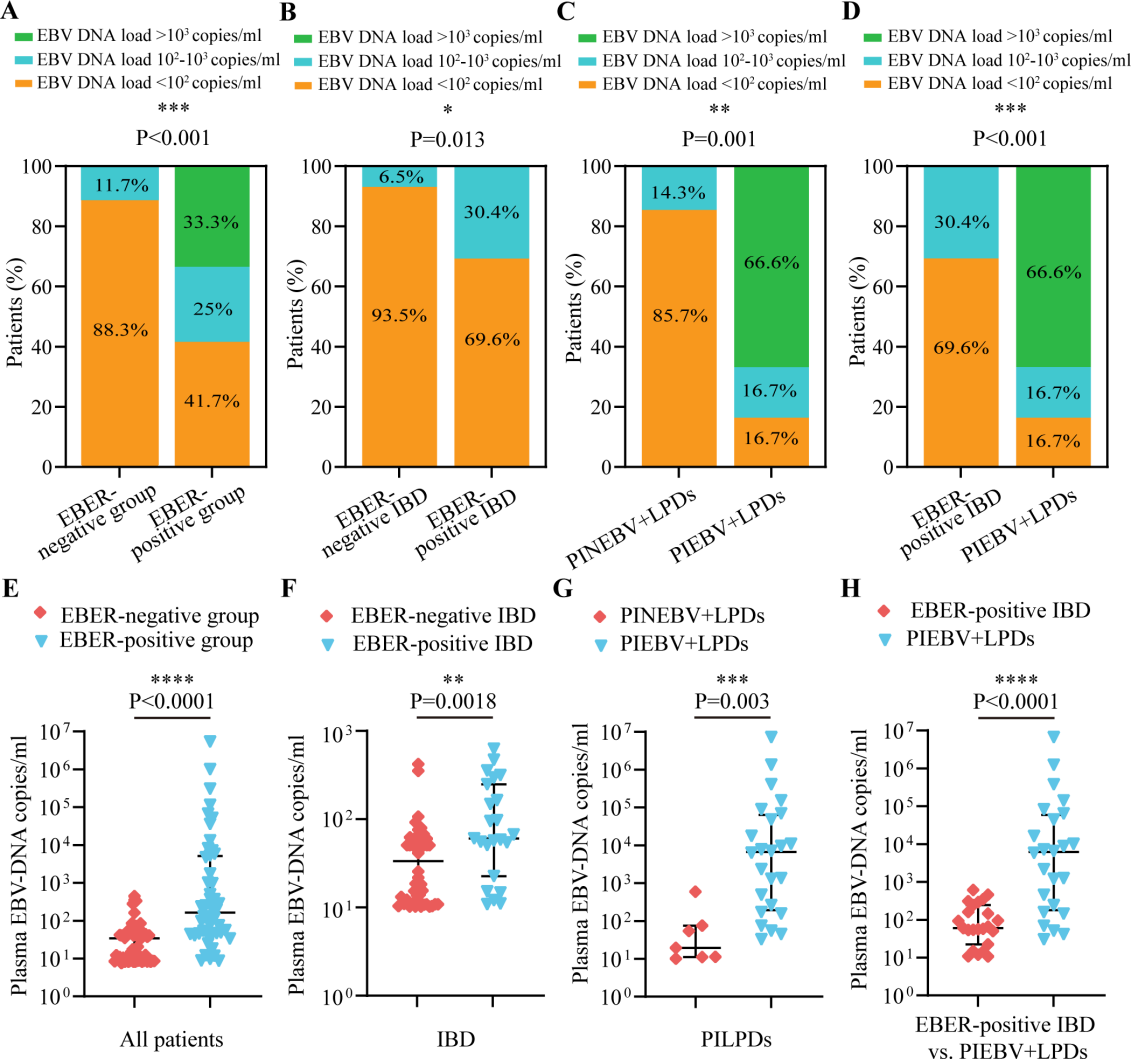
Comparisons of the distribution and quantification of plasma EBV DNA load between EBER-negative and EBER-positive groups. The comparisons include intestinal EBER-positive group vs. intestinal EBER-negative group **(A, E)**, EBER-negative IBD vs. EBER-positive IBD **(B, F)**, PINEBV+LPDs vs. PIEBV+LPDs **(C, G)**, and EBER-positive IBD vs. PIEBV+LPDs **(D, H)**. *p≤0.05, **p≤0.01, ***p≤0.001, and ****p≤0.0001.

In this study, we conducted comparative analyses of the distribution of plasma EBV DNA load across various intestinal diseases. The findings revealed that the majority of patients (88.3%) in the EBER-negative group had EBV DNA loads <10^1^ copies/ml, with only 11.7% having loads of 10^2^-10^3^ copies/ml, whereas the EBER-positive group exhibited a wider distribution of higher plasma EBV DNA loads, with 33.3% having loads >10^3^ copies/ml and 25% having loads between 10^2^ and 10^3^ copies/ml (*P* < 0.001; **Figure 1A**). Additionally, when focusing on patients with IBD, we observed that EBER-positive IBD patients exhibited a notably elevated proportion of plasma EBV DNA loads (10^2^-10^3^ copies/ml) compared to their EBER-negative counterparts (30.4% vs. 6.5%, *P* = 0.013; **Figure 1B**). Among patients with PILPDs, 66.6% of PIEBV+LPD patients had EBV DNA loads >10^3^ copies/ml, whereas no patients in the PINEBV+LPD group exhibited similarly high viral loads (*P* = 0.001; **Figure 1C**). Despite the overall high occurrence of elevated plasma EBV DNA loads in the EBER-positive IBD and PIEBV+LPD groups, a notable difference was observed in the distribution of viral loads between these two groups. In the PIEBV+LPD group, 66.6% of the patients had EBV DNA loads >10^3^ copies/ml, whereas none of the EBER-positive IBD patients were present within this viral load range (*P* < 0.001; **Figure 1D**).

Further analysis was conducted to compare plasma EBV DNA levels across EBER-negative and EBER-positive populations. Among the 48 patients in the intestinal EBER-positive group, the plasma EBV DNA level had a median value of 2.02 × 10^2^ copies/mL (Interquartile Range [IQR]: 5.49×10^1^-6.34×10^3^ copies/mL), which represented a significantly elevated level compared to the median of 4.2×10¹ copies/mL (IQR: 1.07×10^1^-6.08×10^1^ copies/mL) observed in 60 patients in the intestinal EBER-negative group (**Figure 1E**). These median plasma EBV DNA loads were notably elevated in comparison to the respective EBER-negative control groups, including the IBD and PILPD groups, as detailed in **Figures 1F, G**. Notably, within the intestinal EBER-positive cohort, patients diagnosed with PIEBV+LPDs demonstrated the highest median plasma EBV DNA level at 6.18×10^3^ copies/mL (IQR 1.8×10^2^-5.91×10^4^ copies/mL), significantly surpassing the levels in EBER-positive IBD patients (5.96×10^1^ copies/mL [IQR 2.23×10^1^-2.45×10^2^ copies/mL]), with a *P*-value of < 0.0001 (**Figure 1H**).

### 3.3 Correlation analysis of EBER-positive cell counts with plasma EBV DNA levels in intestinal EBV infection

**Figure 2:**
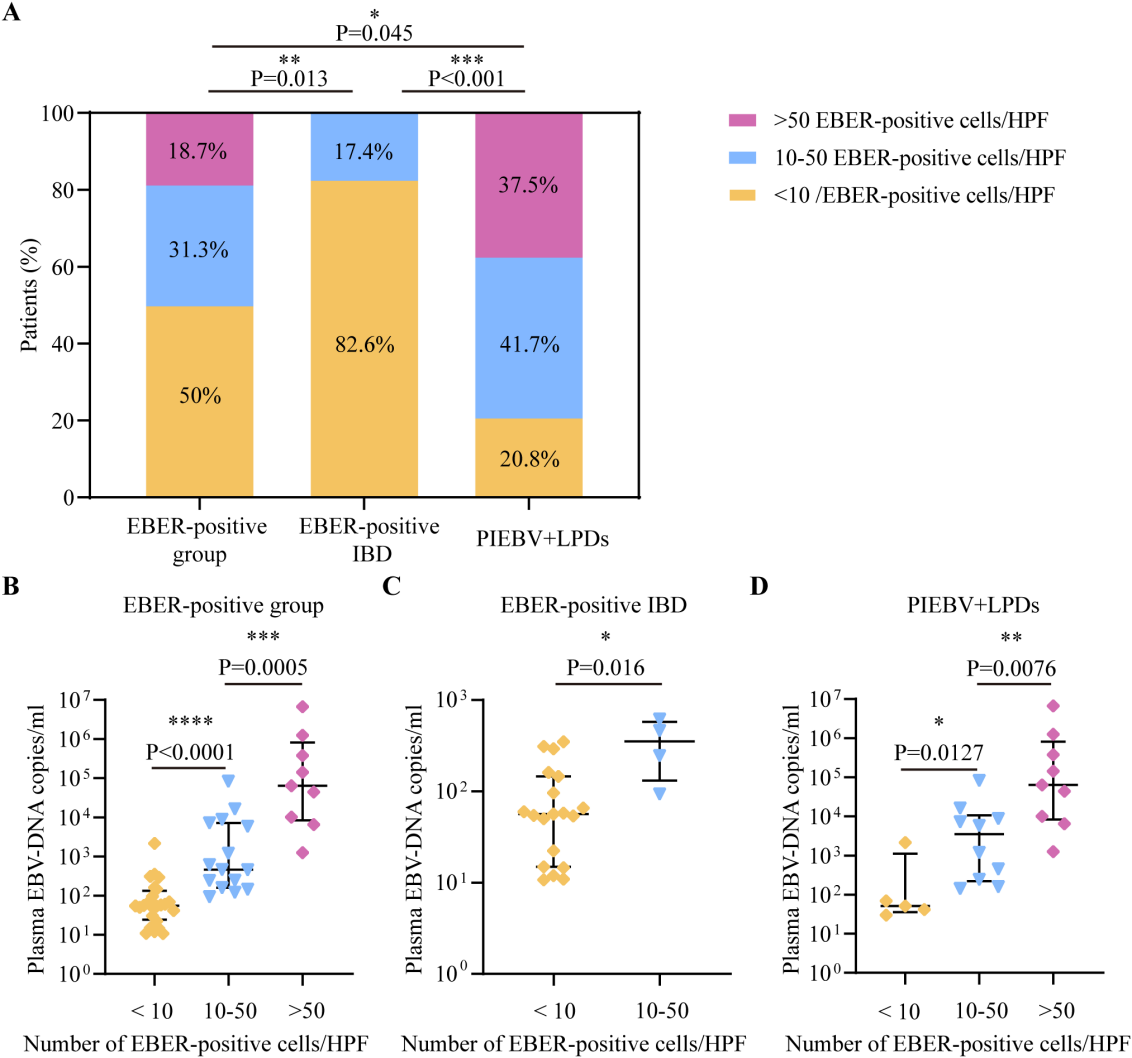
Comparative analysis of EBV DNA concentrations in plasma in accordance with total count of cells positive for EBER per HPF in intestinal tissue. **(A)** Distribution of total cells positive for EBER-ISH per HPF in intestinal tissue across different intestinal diseases with EBV infection. **(B)** Plasma EBV DNA quantity within EBER-positive patients. **(C)** Plasma EBV DNA quantity in EBER-positive IBD. **(D)** Plasma EBV-DNA quantity in PIEBV+LPDs. *p≤0.05, **p≤0.01, ***p≤0.001, and ****p≤0.0001.

According to the consensus established on tissue identification and diagnostic pathology of intestinal EBV infection from the Chinese Medical Association (Ye et al., 2019), we divided these patients into three categories based on the number of cells positive for EBER-ISH per HPF: <10 cells per HPF, 10-50 cells per HPF, and >50 cells per HPF. We analyzed the interrelationship between plasma EBV DNA load and the frequency of intestinal EBER-positive cells per HPF in the intestinal EBER-positive cohort and found that the largest segment comprised patients with <10 cells positive for EBER-ISH per HPF (50%), followed by those with 10-50 cells positive for EBER-ISH per HPF (31.3%), and then patients with >50 cells positive for EBER-ISH per HPF (18.7%; **Figure 2A**). Notably, there was a notable disparity between PIEBV+LPDs and EBER-positive IBD, with 79.2% of PIEBV+LPD patients exhibiting more than 10 cells positive for EBER-ISH per HPF, compared to only 17.4% in the EBER-positive IBD group (*P* < 0.001; **Figure 2A**).

For a profound evaluation of EBV DNA load in plasma within intestinal EBV infection, we assessed the median plasma EBV DNA concentrations across different count ranges of EBER-positive cells per HPF, and we discovered that the median plasma DNA concentrations of EBV in the group with >50 EBER-positive cells per HPF (6.41×10^4^copies/mL) and in the group with 10-50 EBER-positive cells per HPF (4.62×10^2^ copies/mL) were both markedly elevated compared to the level within the group with <10 EBER-positive cells per HPF (5.53×10^1^ copies/mL), as shown in **Figure 2B**. Similarly, within the EBER-positive IBD subgroup, patients with 10-50 EBER-positive cells per HPF demonstrated significantly elevated plasma EBV DNA levels compared to those with <10 EBER-positive cells per HPF (3.54×10^2^ copies/mL vs. 5.62×10^1^ copies/mL; **Figure 2C**). Furthermore, in PIEBV+LPD group, patients with >50 EBER-positive cells per HPF exhibited markedly higher plasma EBV DNA levels (median 6.41×10^4^ copies/mL), which were much superior in comparison with those with 10-50 EBER-positive cells per HPF (median 3.52×10^3^ copies/mL, *P* = 0.0076) and those with <10 EBER-positive cells per HPF (median 5.09×10^1^ copies/mL, *P* = 0.0127), as delineated in **Figure 2D**.

### 3.4 Diagnostic implication of plasma EBV DNA quantification in intestinal EBV infection

**Figure 3:**
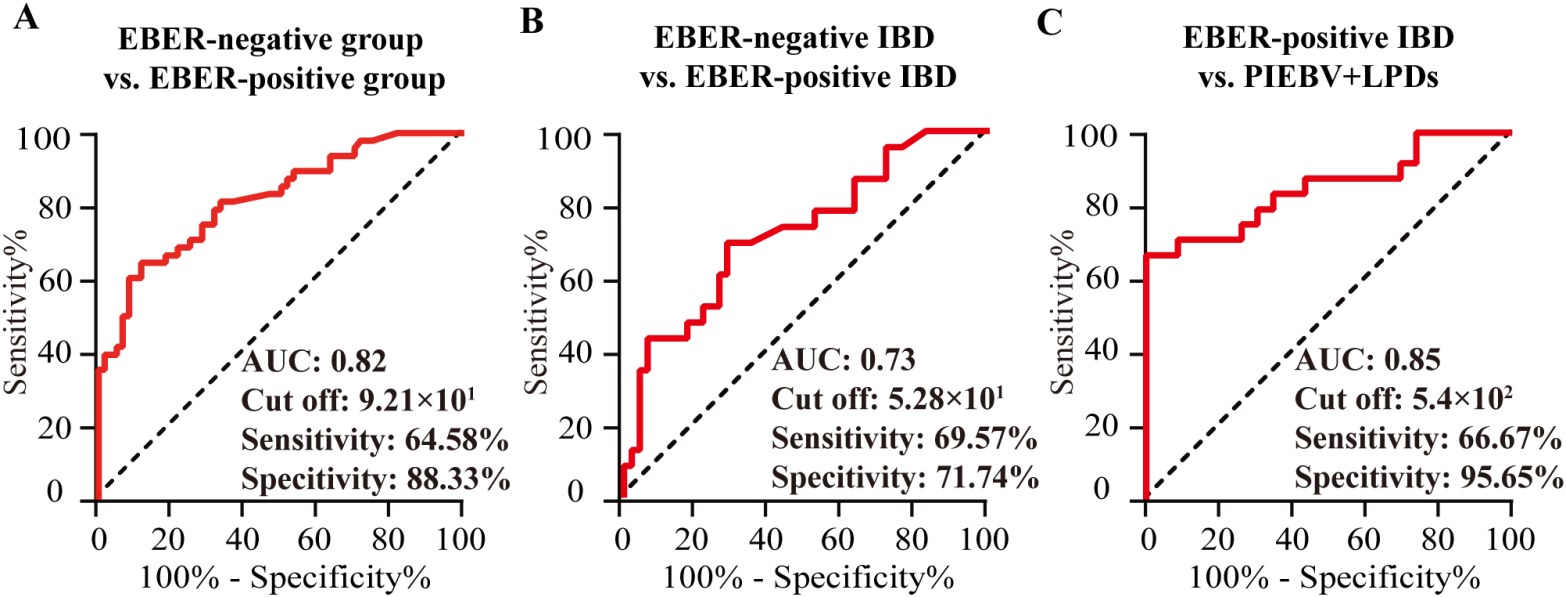
Analysis in the test cohort to assess the diagnostic utility of plasma EBV DNA quantification. **(A)** EBER-negative vs. EBER-positive group; **(B)** EBER-negative vs. EBER-positive IBD. **(C)** EBER-positive IBD vs. PIEBV+LPDs.

Considering that the density of intestinal cells positive for EBER-ISH per HPF exhibited a positive correlation with plasma EBV DNA levels, receiver operating characteristic (ROC) curve examination was initiated to ascertain the potency of plasma EBV DNA load for screening intestinal EBV infection in the test cohort. The analysis indicated that the area under the ROC curve (AUC) for distinguishing intestinal EBER positivity from EBER negativity was 0.82 at a concentration of 9.21×10^1^ copies/mL of EBV DNA in plasma (**Figure 3A**), with a sensitivity of 64.58% and a specificity of 88.33%, indicating its potential utility in initial screening for EBV status. Furthermore, subgroup analyses stratified by intestinal EBER-ISH status identified 5.28×10^1^ copies/mL as the cutoff value for differentiating EBV infection from non-EBV infection in IBD, yielding an AUC of 0.73 (**Figure 3B**), with a sensitivity of 69.57% and a specificity of 71.74%. This result suggests that plasma EBV DNA load has moderate discriminatory power in distinguishing EBV-positive IBD from EBV-negative IBD. Additionally, the analysis was extended to differentiate between EBER-positive IBD and PIEBV+LPDs. The ROC curve analysis for these two diseases produced an AUC of 0.85, with a discriminative threshold established at 5.4×10^2^ copies/mL with high specificity (95.65%) to ensure diagnostic accuracy for this rare but severe condition (**Figure 3C**).

The detection capacity achieved by plasma EBV DNA cutoff values indicating intestinal EBV infection was evaluated in the validation cohort. Analysis of clinical characteristics revealed no significant differences in demographic features and clinical presentations within the test and validation cohorts (**Supplementary Table S1**). In the validation cohort, a threshold of 9.21×10^1^ copies/mL for EBV DNA load in plasma demonstrated an LR^+^ calculated at 5.06, attaining values of 83.33%, 84.38%, 80%, and 87.1% in terms of specificity, sensitivity, NPV, and PPV, respectively, for identifying intestinal EBER-positive and EBER-negative diseases (**Table 2**). For IBD, the sensitivity, specificity, PPV, NPV, and LR^+^ of a threshold of 5.28×10^1^ copies/mL to differentiate intestinal EBV infection from non-EBV infection were 66.67%, 55.56%, 50%, 71.43%, and 1.5, respectively (**Table 2**). Furthermore, at a cutoff value of 5.4×10^2^ copies/mL, the sensitivity for distinguishing PIEBV+LPDs from EBER-positive IBD reached 85%, with an LR^+^ of 10.2 (**Table 2**).

**Table 2:** Detection capacity of plasma EBV DNA cutoff values for intestinal EBV infection within the validation subset.

Cut off values of plasma EBV DNA load (copies/ml)	Sensitivity (%) (95%CI)	Specificity (%) (95%CI)	PPV (%) (95%CI)	NPV (%) (95%CI)	LR^+^	LR^-^
9.21×10^1^	84.38 (68.25, 93.14)	83.33 (64.15, 93.32)	87.1 (71.15, 94.87)	80 (60.87, 91.14)	5.06	0.19
5.28×10^1^	66.67 (39.06, 86.19)	55.56 (33.72, 75.44)	50 (28, 72)	71.43 (45.35, 88,28)	1.5	0.6
5.4×10^2^	85 (63.96, 94.76)	91.67 (64.61, 99.57)	94.44 (74.24, 99.72)	78.57 (52.41, 92.43)	10.2	0.16

LR^-^, negative likelihood ratio; NPV, negative predictive value; LR^+^, positive likelihood ratio; PPV, positive predictive value; 95%CI: 95% confidence interval.

### 3.5 Prognostic implication of plasma EBV DNA quantification in intestinal EBV infection

The analysis further explored and compared the six-month prognosis following intestinal EBER-ISH test among patients with intestinal diseases who were positive for EBV DNA load in plasma. The results confirmed that individuals in the intestinal EBER-positive group displayed significantly poorer prognoses than those in the EBER-negative group of the test cohort (50% vs. 15%, *P* < 0.001). Moreover, EBER-positive IBD and PIEBV+LPD patients demonstrated worse outcomes than their EBER-negative counterparts (26.1% vs. 15.2%, *P* =0.334 and 75% vs. 28.6%, *P* = 0.067, respectively). Details comparing the prognostic outcomes of various intestinal diseases are presented in **Supplementary Table 2**.

**Figure 4:**
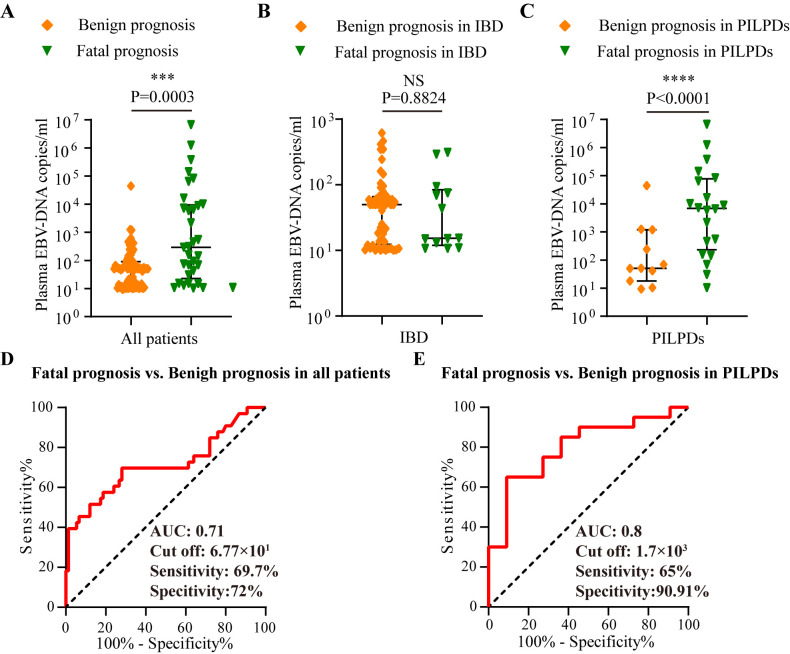
Analysis in the test cohort to assess the prognostic utility of plasma EBV DNA quantification. Comparative analysis of plasma EBV-DNA concentrations among various prognosis within the test cohort, including **(A)** Overall cohort, **(B)** IBD cohort, and **(C)** PILPD cohort. **(D)** ROC curve comparing fatal versus benign prognoses in all patients. **(E)** ROC curve comparing the fatal and benign prognoses in patients with PILPDs. ***p < 0.001, ****p < 0.0001. NS, not significant.

Comparative analyses of plasma EBV DNA concentrations between the fatal and benign groups with various intestinal diseases were also conducted. As depicted in **Figure 4A**, patients with a fatal prognosis had greater median plasma EBV DNA loads than those with a benign outcome (2.9×10^2^ copies/mL vs. 5.02×10^1^ copies/mL, *P* = 0.0003). Furthermore, patients with PIEBV+LPDs and those with PINEBV+LPDs had significantly different median plasma EBV DNA levels (6.86×10^2^ copies/mL vs. 5.09×10^1^ copies/mL, *P* < 0.0001, **Figure 4C**). However, patients with fatal prognosis did not show a statistically significant difference in plasma EBV DNA load compared to those with benign prognosis in IBD (P=0.8824) (**Figure 4B**).

ROC curve analysis revealed that there was an excellent connection between plasma EBV DNA concentrates and prognosis for intestinal EBV infection, demonstrating an AUC of 0.71 in the test cohort (**Figure 4D**), with a sensitivity of 69.7% and a specificity of 72%, demonstrating moderate ability to differentiate between fatal and benign prognoses. By establishing a threshold of 6.77×10^1^ copies/mL for plasma EBV DNA load, we established the sensitivity, specificity, PPV, NPV, LR^+^, and LR^-^ for differentiating between fatal and benign prognoses in the validation cohort. The resulting values were 87.5%, 68.09%, 58.33%, 91.43%, 2.74, and 0.18, respectively (**Table 3**). Furthermore, plasma EBV DNA concentration at 1.7×10^3^ copies/mL was shown to be the most effective threshold for predicting PILPD prognosis, with an AUC of 0.8 (**Figure 4E**), with a high specificity of 90.91% and relatively low sensitivity of 65%, ensuring the accurate identification of patients at risk of severe outcomes of PILPDs. Within the validation subset, the established cutoff offered a sensitivity of 64.71%, specificity of 83.33%, PPV of 91.67%, NPV of 45.45%, LR^+^ of 3.88, and LR^-^ of 0.42, effectively distinguishing between benign and fatal outcomes in PILPDs (**Table 3**).

**Table 3:** The ability to evaluate prognostic outcomes based on plasma EBV DNA cutoff values for intestinal EBV infection within the validation subset.

Cut off values of plasma EBV DNA load (copies/ml)	Sensitivity (%) (95%CI)	Specificity (%) (95%CI)	PPV (%) (95%CI)	NPV (%) (95%CI)	LR^+^	LR^-^
6.77×10^1^	87.5 (69, 95.66)	68.09 (53.83, 79.6)	58.33 (42.2, 72.86)	91.43 (77.62, 97.04)	2.74	0.18
1.7×10^3^	64.71 (41.3, 82.69)	83.33 (43.65, 99.15)	91.67 (64.61, 99.57)	45.45 (21.27, 71.99)	3.88	0.42

LR^-^, negative likelihood ratio; NPV, negative predictive value; LR^+^, positive likelihood ratio; PPV, positive predictive value; 95%CI: 95% confidence interval.

## 4 Discussion

This investigation found a positive alignment between plasma EBV DNA quantification and the number of cells positive for EBER-ISH per HPF in intestinal diseases. Further studies were performed to determine the significance of EBV DNA concentrations in plasma with respect to identifying intestinal EBV infection and predicting its outcome, and specific threshold values were established. These results indicate that the EBV DNA quantity in plasma functions as a credible marker for the diagnostic and prognostic assessment of intestinal EBV infection.

Emphasizing clinical characteristics plays a crucial role in identifying EBV infection. Our study found no difference in immunosuppressant use between infected and non-infected patients, whereas previous studies have identified immunosuppressant administration as a pivotal element in triggering intestinal EBV activation (Ford and Peyrin-Biroulet, 2013; Magro et al., 2013; Lapsia et al., 2016). This may be due to the fact that all participants in both cohorts of this study were patients with detectable plasma EBV DNA, indicating that both groups were already in a state of EBV activation, which could mask the potential impact of immunosuppressant use. Research has shown that the main clinical manifestations of primary EBV infection include fever, pharyngitis, lymphadenopathy, and hepatosplenomegaly (Song et al., 2023; Ye et al., 2023). Consistent with this, our research demonstrated that compared to those who did not have EBV infection in their intestines, those with intestinal EBV infection exhibited fever symptoms more frequently. Moreover, our investigation is the first cohort study to affirm that patients with intestinal EBV infection strongly exhibit a higher predisposition to gastrointestinal manifestations with hematochezia than those without infection, which aligns with sporadic reports of primary intestinal EBV infection (Karlitz et al., 2011; Chen et al., 2016; Wang Y. et al., 2018). Therefore, our study suggests that it is essential to raise clinical vigilance in screening for intestinal EBV infection in patients with gastrointestinal complaints and fever symptoms.

In general, EBV maintains latency within a robust immune system. However, immunodeficiencies triggered by many factors can result in the reactivation of EBV (Chan et al., 2021). In such scenarios, a detectable level of EBV DNA signifies that the virus is actively undergoing infection and replication, whereas the histological EBER-ISH test is used to ascertain the presence of EBV in tissue samples (Kanakry et al., 2013; Liang et al., 2015; Zhou et al., 2020a). In this study, a substantial increase in the detection of plasma EBV DNA was observed among patients suffering from intestinal EBV infection, in contrast to those who were unaffected. The median plasma EBV DNA loads were reported as 2.02×10^2^ copies/ml in infected patients versus 4.2×10^1^ copies/ml in non-infected individuals, corroborating the findings of prior studies on EBV-associated diseases (Yu et al., 2021; Shen et al., 2022). This discrepancy indicates that patients with intestinal EBV infection typically experience persistent viral replication, leading to elevated plasma viral loads. Furthermore, consistent with earlier studies (Zhou et al., 2020b; Wang et al., 2022), our study found that IBD patients with intestinal EBV infection showed higher plasma EBV DNA levels than those not infected. Even though the value of EBV DNA load varies across studies owing to the different samples and methods used for EBV DNA testing, the trends observed in these studies are consistent.

The results of our study indicate that plasma EBV DNA load is positively correlated with the number of intestinal EBER-positive cells per HPF. This is consistent with the findings reported by Zhou et al. (Zhou et al., 2020b) in IBD patients with intestinal EBV infection, and aligns with the results from other studies related to EBV-associated diseases (Kanakry et al., 2016; Yu et al., 2021; Shen et al., 2022). Moreover, an indicator of intestinal EBER positivity based on plasma EBV DNA levels was examined using ROC analysis, revealing an AUC of 0.82. The optimal cut-off was established at 9.21×10^1^ copies/ml, which is sensitive to 64.58% and specific to 88.33% in distinguishing intestinal EBV infection from non-infection. This is consistent with a previous study that used detectable EBV DNA to segregate EBV-associated diseases from those unrelated to EBV, exhibiting a sensitivity of 83.9% and specificity of 93.5% (Yu et al., 2021). It is evident that our investigation proposed an EBV DNA cut-off value with greater precision for diagnosing intestinal EBV infection. Moreover, our study revealed that patients with IBD with intestinal EBV infection can be distinguished from those with PIEBV+LPDs when their plasma EBV DNA concentration is below 5.4×10^2^ copies/ml, which contributes to the differential diagnosis of the two diseases. Hence, our study demonstrates that it is possible to use plasma DNA load as a screening and monitoring tool for intestinal EBV infection. Nevertheless, it is vital to recognize that histological EBER-ISH test might yield underestimations of EBV infection because of the minimal thickness of tissue sections, whereas the high sensitivity of PCR technology in detecting EBV DNA might lead to overestimation of EBV infection. Furthermore, in clinical practice, IBD with intestinal EBV infection is generally regarded as an opportunistic infection, whereas PIEBV+LPDs represent conditions in which EBV acts as a direct pathogenic factor (Ye et al., 2019). This fundamental difference explains the distinct cutoff values derived for each cohort in this study, reflecting their unique disease contexts. Therefore, selecting an appropriate test method and cutoff value of plasma EBV DNA load, based on real-world clinical needs, is essential to achieve optimal diagnostic performance and prognostic assessment for intestinal EBV infection, such as improving the detection accuracy of EBV infection in IBD patients or ensuring precise differentiation in PIEBV+LPDs.

The heterogeneity of outcomes associated with EBV infection spans a continuum from benign to malignant manifestations, leading to prognostic variability in EBV-associated diseases. Prior studies have demonstrated plasma EBV DNA load for predicting the outcome and therapeutic response of EBV-associated diseases (Kanakry et al., 2013; Ito et al., 2016). In cases of intestinal EBV infection, EBV infection has been linked to refractory responses and the necessity for surgical treatment in patients with IBD (Hosomi et al., 2018; Pezhouh et al., 2018). Analysis of a cohort of 12 patients with PIEBV+LPDs revealed a 50% mortality rate within a follow-up interval of 1-21 months (Wang Z. et al., 2018). Our investigation contributes to this body of evidence by demonstrating a significant correlation between elevated plasma EBV DNA levels and fatal prognoses in patients with intestinal EBV infection within six months after the intestinal EBER-ISH test, marked by an AUC of 0.71. Additionally, for patients with PILPDs, when the plasma EBV DNA load exceeds 1.7×10^3^ copies/ml, there is a significant increase in the occurrence of severe adverse outcomes, with an AUC of 0.8. These results further confirm the utility of monitoring plasma EBV DNA load as an indicator of poor outcomes in patients with intestinal EBV-infected infection.

This is an initial investigation to assess the EBV DNA load in plasma as a diagnostic and prognostic tool for intestinal EBV infection. This noninvasive approach promises to diminish the reliance on invasive diagnostic interventions for patients with intestinal EBV infection during the initial diagnosis and subsequent follow-up and holds particular significance for diagnosing and differentiating intestinal diseases associated with EBV. Nonetheless, our study has several limitations. First, our research did not explore the diagnostic and prognostic utility of other types of blood samples, such as whole blood or PBMCs, for intestinal EBV infection. This omission leaves a gap in our understanding of the optimal blood sample type for the accurate diagnosis and prognosis of intestinal EBV infection. Future studies should address this gap by comparing the effectiveness of EBV DNA quantification in different types of blood samples. Second, the retrospective, single-center nature of our study, coupled with a limited sample size, introduces potential biases and limits the generalizability of our findings. To mitigate these limitations and validate the proposed cut-off values for plasma EBV DNA levels, extensive multicenter studies with larger cohorts are essential. Third, the sensitivity of our study was relatively low. To address this limitation, future studies should incorporate additional biomarkers, such as EBV antibody levels and inflammatory markers, as well as other factors, including clinical characteristics and histopathological features, which could collectively enhance the overall diagnostic and prognostic accuracy of plasma EBV DNA load in intestinal EBV infection. Lastly, IBD and PILPD patients constituted the majority of the subjects in this study. Thus, our data do not rule out intestinal EBV infection in patients with other EBV-associated diseases who did not undergo colonoscopy for intestinal EBER-ISH. Studies on a greater variety of study populations with other EBV-associated diseases are needed to evaluate intestinal EBV infection.

The conclusions drawn from this investigation reinforce the importance of plasma EBV DNA quantification in the detection and outcome prediction of intestinal EBV infection. Specifically, the cutoff values of EBV DNA in the plasma at 9.21×10^1^ and 6.77×10^1^ copies/mL facilitate the diagnosis and prognosis of this disease, respectively. This noninvasive method provides robust evidence for the management of intestinal EBV infection. Detection of EBV DNA using various blood samples and larger-scale multicenter studies will be pivotal in advancing the clinical use of EBV DNA quantification in peripheral blood for managing intestinal EBV infection.

## Data Availability

The raw data supporting the conclusions of this article will be made available by the authors, without undue reservation.
