# Supplementary material for: Plasma Epstein-Barr Virus DNA load for diagnostic and prognostic assessment in intestinal Epstein-Barr Virus infection
Source: Front Cell Infect Microbiol. 2025 Jan 7;14:1526633. doi: 10.3389/fcimb.2024.1526633 (PMC11747383; doi:10.3389/fcimb.2024.1526633)

Supplementary Material

# Supplementary Table 1. Clinical characteristics of patients in the test and validation cohorts.

| **Characteristics** | **Test cohort**  **(n=108)** | **Validation cohort**  **(n=56)** | ***P* value** |
| --- | --- | --- | --- |
| Age (year), median ±SD | 43±16 | 44±16 | 0.69 |
| Male, n (%) | 68 (63%) | 36 (64.3%) | 0.868 |
| Disease duration (months), median ±SD | 42±67 | 32±53 | 0.35 |
| IMM use within three months prior to intestinal EBER-ISH test, n (%) | 28 (25.9%) | 13 (23.2%) | 0.704 |
| IBD | 69 (63.9%) | 30 (53.6%) | 0.274 |
| PILPDs | 31 (28.7%) | 23 (41.1%) |  |
| Other diseases | 8 (7.4%) | 3 (5.4%) |  |
| Fever | 36 (33.3%) | 27 (48.2%) | 0.063 |
| Abdominal pain | 79 (73.1%) | 38 (67.9%) | 0.477 |
| Diarrhea | 63 (58.3%) | 33 (58.9%) | 0.942 |
| Haematochezia | 66 (61.1%) | 39 (69.6%) | 0.28 |
| Weight loss | 66 (61.1%) | 30 (53.6%) | 0.353 |

SD, standard deviation; IMM, immunomodulators.

# Supplementary Table 2. The prognosis of patients in the test and validation cohorts.

| **Groups** | **Test cohort** | | | **Validation cohort** | | |
| --- | --- | --- | --- | --- | --- | --- |
|  | **Benign prognosis, n (%)** | **Fatal prognosis, n (%)** | ***P* value** | **Benign prognosis, n (%)** | **Fatal prognosis, n (%)** | ***P* value** |
| EBER-negative group | 51 (85%) | 9 (15%) | <0.001 | 18 (75%) | 6 (25%) | 0.019 |
| EBER-positive group | 24 (50%) | 24 (50%) |  | 14 (43.8%) | 18 (56.3%) |  |
| EBER-negative IBD | 39 (84.8%) | 7 (15.2%) | 0.334 | 13 (72.2%) | 5 (27.8%) | 0.669 |
| EBER-positive IBD | 17 (73.9%) | 6 (26.1%) |  | 10 (83.3%) | 2 (16.7%) |  |
| PINEBV+LPDs | 5 (71.4%) | 2 (28.6%) | 0.067 | 2 (66.7%) | 1(33.3%) | 0.155 |
| PIEBV+LPDs | 6 (25%) | 18 (75%) |  | 4 (20%) | 16 (80%) |  |

# Supplementary Figure 1. Diagram of the criteria for patient selection and exclusion.


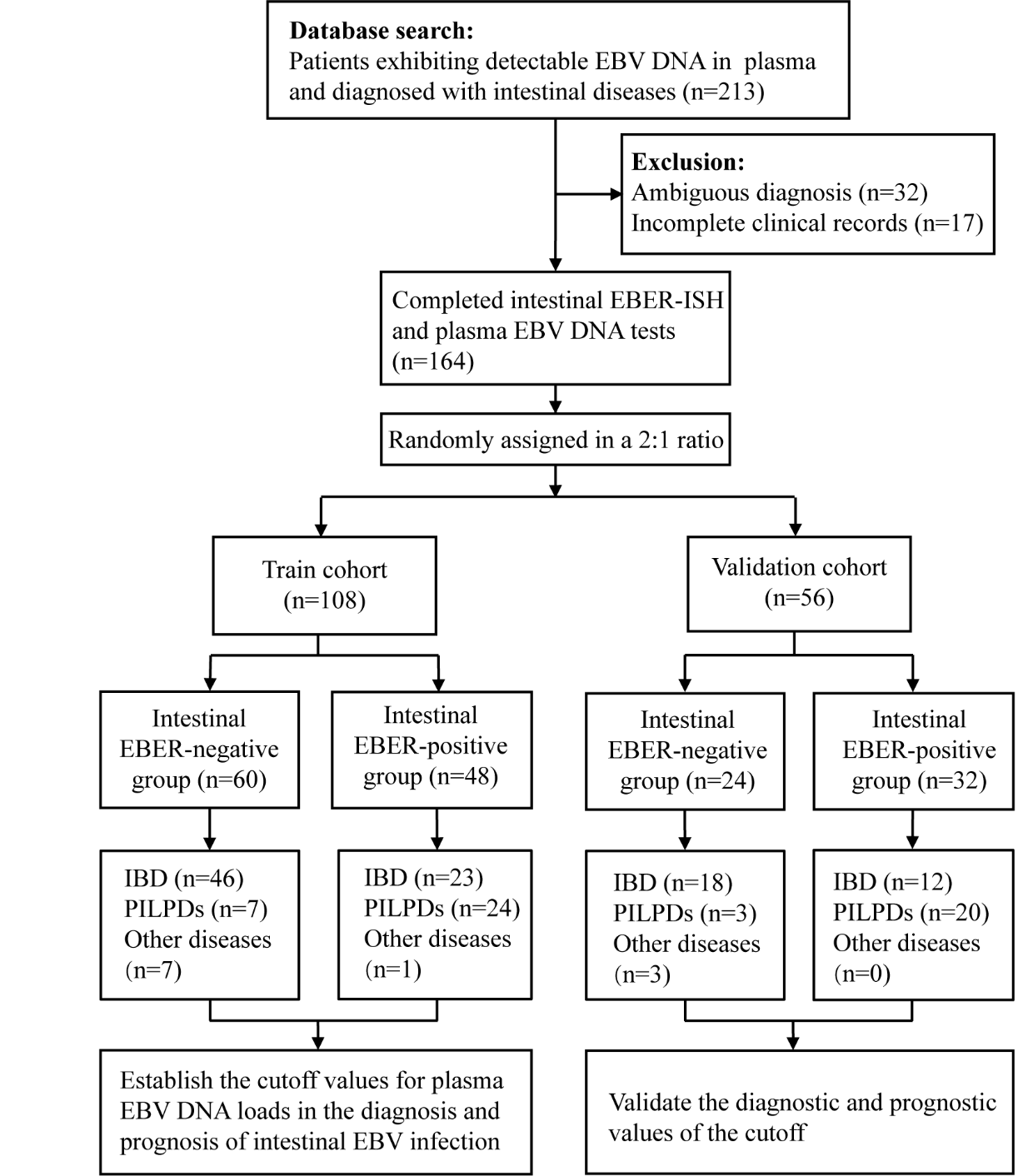

Supplement: Supplementary file 1 [file Table1.docx]
